# The Role of Sodium-Glucose Co-transporter 2 Inhibitors in Patients With Hypomagnesemia: A Systematic Review

**DOI:** 10.7759/cureus.60919

**Published:** 2024-05-23

**Authors:** Madhusudan P Singh, Nikunj Agrawal, Abhimanyu Agrawal, Salil S Kushwah, Ekta Krishna, Juhi M Singh

**Affiliations:** 1 Pharmacology and Therapeutics, All India Institute of Medical Sciences, Raipur, Raipur, IND; 2 General Medicine, Laxmi Narayan Medical College, Bhopal, IND; 3 Pediatrics, Bal Gopal Children Hospital, Raipur, IND; 4 Community and Family Medicine, All India Institute of Medical Sciences, Patna, Patna, IND; 5 Pathology, Kanti Devi Medical College, Mathura, IND

**Keywords:** magnesium homeostasis, refractory hypomagnesemia, type 2 diabetes, hypomagnesemia, sodium-glucose cotransporter 2 (sglt2) inhibitors

## Abstract

Sodium-glucose co-transporter 2 (SGLT2) inhibitors, initially developed for glycemic control in type 2 diabetes, have demonstrated benefits in reducing heart failure hospitalizations, slowing chronic kidney disease, and decreasing major cardiovascular events. Recent studies have shown that SGLT2 inhibitors can elevate serum magnesium levels in patients with type 2 diabetes, suggesting potential benefits in managing refractory hypomagnesemia.

This systematic review analyzed relevant case reports, observational studies, and randomized controlled trials (RCTs) to investigate the association between SGLT2 inhibitors and hypomagnesemia. The review adhered to Preferred Reporting Items for Systematic Reviews and Meta-Analyses (PRISMA) guidelines, and study quality was assessed using the CAse REport (CARE) guidelines. It encompassed four case reports, one retrospective observational study, one post-hoc analysis of 10 RCTs, and one meta-analysis of 18 RCTs, with a total study population of 19,767 patients.

The meta-analysis revealed that SGLT2 inhibitors significantly increased serum magnesium levels in patients with type 2 diabetes, with a linear dose-dependent increase noted particularly for canagliflozin. Additionally, the case reports and other studies suggested that SGLT2 inhibitors could exert extraglycemic effects, potentially enhancing magnesium balance beyond their impact on urinary magnesium excretion.

This systematic review underscores the effectiveness of SGLT2 inhibitors in addressing refractory hypomagnesemia linked with urinary magnesium wasting. It also suggests promising avenues for the application of these drugs in diverse patient populations.

## Introduction and background

Sodium-glucose co-transporter 2 inhibitors (SGLT2i) have emerged as a crucial treatment for diabetes mellitus type 2, offering benefits beyond blood sugar control [1]. Clinical trials have demonstrated their effectiveness in reducing hospitalizations due to heart failure, slowing the progression of chronic kidney disease, and decreasing major cardiovascular events, with consistently lower mortality rates [1]. Recent evidence suggests a class effect of SGLT2 inhibitors, with randomized controlled studies revealing a significant elevation in serum magnesium levels, ranging from 0.04-0.1 mmol/L, in patients with type 2 diabetes, regardless of baseline hypomagnesemia [2,3]. However, the role of SGLT2 inhibitors in treating hypomagnesemia in non-diabetic patients remains unexplored.

Magnesium, a crucial element involved in over 600 enzymatic reactions in the human body, plays a vital role in maintaining the stability of nucleic acids and nucleotide triphosphates, as well as modulating cellular electrical activity [4]. Magnesium depletion can manifest in a wide range of clinical effects, impacting cardiovascular, neuropsychiatric, and endocrine functions, with particular relevance to conditions like type 2 diabetes [5]. Managing severe hypomagnesemia (normal level: 1.6-2.5 mg/dl) presents significant challenges. Gastrointestinal absorption limitations hinder the effectiveness of oral supplementation, while intravenous magnesium infusions provide only temporary increases in serum magnesium levels due to enhanced urinary excretion [6]. For individuals with urinary magnesium wasting disorders, persistent hypomagnesemia often persists, further complicating treatment strategies [7].

The potential link between hypomagnesemia and glucose intolerance, especially in the context of diabetes mellitus, warrants further investigation. SGLT2 inhibitors, established for their role in diabetes management, are associated with a significant increase in serum magnesium concentration among diabetic patients [2]. This intriguing connection highlights the potential of SGLT2 inhibitors not only for glycemic control, but also as contributors to magnesium balance. This finding offers promise in addressing refractory hypomagnesemia, a condition with limited treatment options, even in patients without diabetes.

This systematic review aims to comprehensively analyze the available literature to explore the association between SGLT2 inhibitors and hypomagnesemia. This review explores the symptoms, treatment options, and patient outcomes associated with hypomagnesemia in individuals undergoing SGLT2 inhibitor therapy. It seeks to provide crucial insights for healthcare professionals.

## Review

Materials and methods

Data Sources and Search Strategy

This systematic review has been conducted following the guidelines outlined in the Preferred Reporting Items for Systematic Reviews and Meta-Analyses (PRISMA) [8]. This search covered major databases like SCOPUS, Clinicaltrials.gov, MEDLINE, Web of Science, and Google Scholar from January 2019 to December 2023. We focused on English language studies and employed a detailed search strategy encompassing various names for SGLT2 inhibitors (pharmaceutical, generic, and brand names). A detailed search string has been provided in the Appendix. To ensure completeness, we also hand-searched the reference lists of the included studies for any additional relevant articles. The comprehensive search strategies for all databases can be found in the Appendix.

Study Selection

Data extraction and assessment of study quality: Following a comprehensive electronic search, all retrieved citations were imported into EndNote reference management software for deduplication. Title and abstract screening were performed independently by two reviewers (Singh and Agrawal) to identify potentially relevant articles. Subsequently, a full-text evaluation of the shortlisted articles was conducted by two additional independent reviewers (Krishna and Kushwah). Any discrepancies arising during the screening process were resolved through adjudication by a third reviewer (Kumari). Data extraction from the included studies encompassed demographic characteristics, co-morbidities, disease onset, initial presentation, laboratory findings, type of diabetes mellitus, specific SGLT2 inhibitor utilized, study publication date, study design, treatment intervention, and reported outcomes. The methodological quality of the included case reports was assessed using the CARE guidelines [9].

Synthesis of results: A narrative synthesis approach was adopted for the qualitative analysis of data extracted from the included case reports. This selection acknowledges the inherent limitations of case reports, such as restricted sample sizes and the absence of control groups. These limitations render the detection of small effect sizes challenging, thereby precluding the implementation of quantitative analysis or the calculation of effect estimates. Consequently, a descriptive synthesis was employed to comprehensively summarize the emergent themes and findings across the incorporated case reports.

Results

Study Results, Study Characteristics, and Baseline Demographics

**Figure 1:**
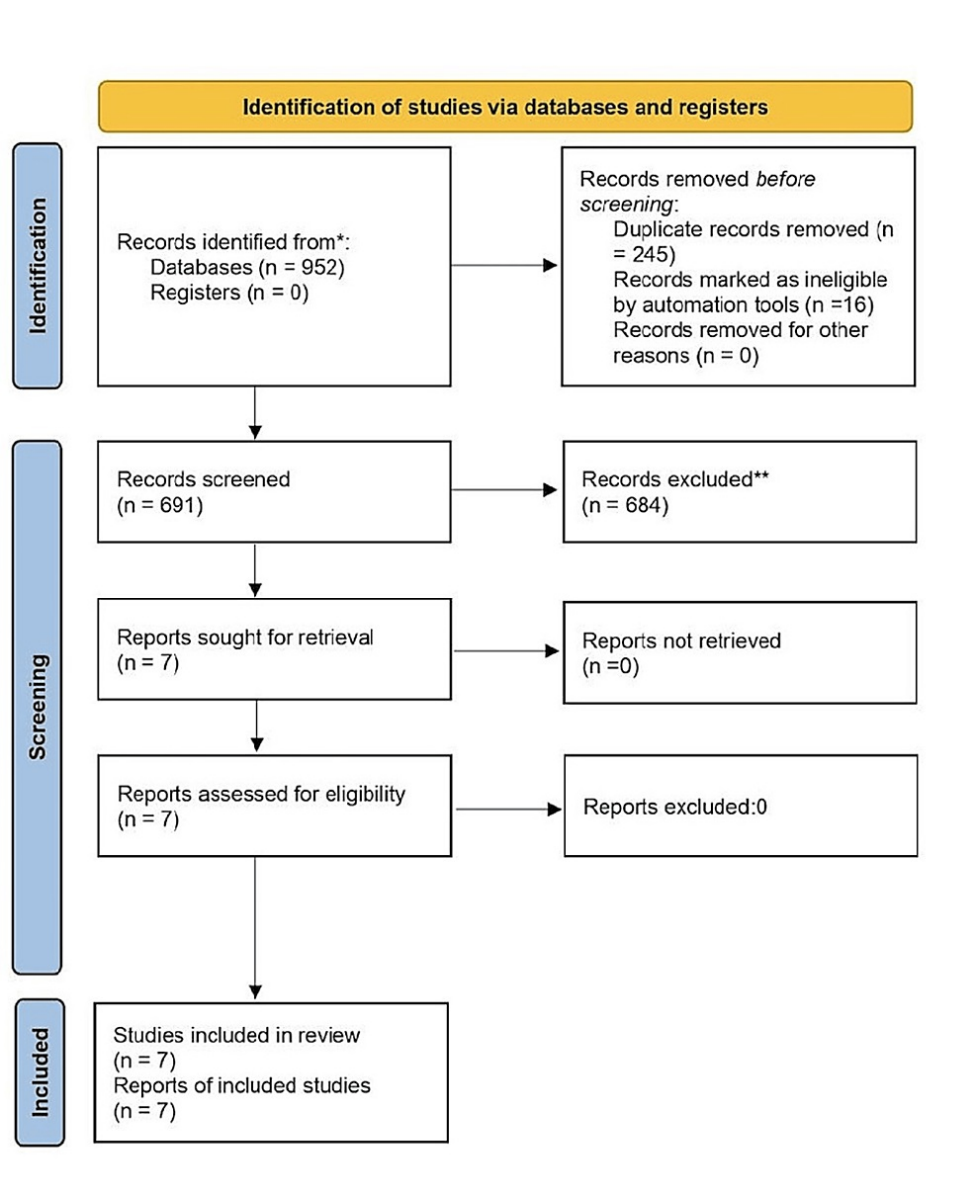
Preferred Reporting Items for Systematic Reviews and Meta-Analyses (PRISMA) Flow Diagram.

The study selection process is illustrated using the PRISMA flow chart (Figure 1), which delineates the search and selection of studies. The initial search across multiple databases yielded a total of 952 studies. The included studies consisted of four case reports, one retrospective observational study, one post-hoc analysis of 10 randomized controlled trials (RCTs), and one meta-analysis. Collectively, the studies encompassed a total of 19,767 patients, with the case reports involving 10 patients, the retrospective study comprising 50 patients, the post-hoc analysis of RCTs including 4,398 patients, and the meta-analysis of 18 RCTs involving a sample size of 15,309 patients.

Quality Assessment

All studies included within this systematic review adhered to extant reporting guidelines, thus ensuring standardized and replicable case reporting. A comprehensive quality assessment table, provided in Appendix (Tables 2, 3) offers a meticulous evaluation of the methodological quality of the included case reports and the observational study.

Evidence Synthesis

The evidence synthesis for this review involved a comprehensive evaluation of the available literature on the impact of SGLT2 inhibitors on serum magnesium levels in patients with type 2 diabetes mellitus (T2DM) and other conditions associated with hypomagnesemia. The meta-analysis conducted by Tang et al. demonstrated that SGLT2 inhibitor administration in patients without chronic kidney disease resulted in a statistically significant elevation of serum magnesium levels compared to placebo control. Notably, canagliflozin exhibited a linear dose-dependent association with increased magnesium levels. Additionally, dapagliflozin was found to significantly increase serum phosphate levels, while changes in serum sodium, potassium, and calcium were not statistically significant.

The included case reports and other studies yielded additional insights into the potential therapeutic utility of SGLT2 inhibitors for managing hypomagnesemia, extending their benefit to patients who may not exhibit overt urinary magnesium wasting. These studies suggested that SGLT2 inhibitors could have extraglycemic effects, potentially improving magnesium balance through mechanisms beyond their impact on urinary magnesium excretion. The post-hoc analysis of RCTs by Toto et al. specifically evaluated the impact of dapagliflozin 10 mg on serum magnesium concentrations in individuals with T2DM. The study reported a higher prevalence of hypomagnesemia in this population and demonstrated that dapagliflozin 10 mg led to a significantly greater mean change in serum magnesium compared to placebo, particularly in patients with baseline hypomagnesemia (Table 1).

**Table 1:** Summary of studies included in the systematic review. DM: diabetes mellitus, Mg: magnesium, T2D: type 2 diabetes mellitus, FBS: fasting blood sugar, FEMg: fractional excretion of magnesium, N.A.: not applicable, AM: ante meridiem, PM: post meridiem, DAPA-HF: dapagliflozin and prevention of adverse outcomes in heart failure, PTH: parathyroid hormone, UTI: urinary tract infection.

Study	Study origin	Study design	No. of patients	Case number	Sex	Mean age (in years)	Type of DM	Serum analysis test pre-SGLT2i	Serum analysis test post-SGLT2i	Urine analysis	Cause of hypomagnesemia	Therapy for hypomagnesemia	SGLT2i used	Outcomes
Shah et al. 2023 [10]	USA	Case report	4	1	Female	50	No DM	FBS (mg/dL): 101; Potassium (mEq/L): 4.4; Calcium (mg/dL): 10.3; Albumin (gm/dL): 4.2; Creatinine (mg/dL): 1.36	FBS (mg/dL): 98; Potassium (mEq/L): 4.4; Calcium (mg/dL): 10.4; Albumin (gm/dL): 4.4; Creatinine (mg/dL): 1.40	24-h urine output (mg): NA.; FEMg: Pre-9.73%; Post-SGLT2-inhibitor-4.88%	Calcineurin inhibitor	Patient was started on oral magnesium 84 mg 4 caplets two times a day (total of 672 mg of elemental magnesium per day)	Empagliflozin 25 mg/d	After the start of empagliflozin, the patient no longer required oral magnesium supplements. Two months after stopping supplements, their magnesium levels remained within normal range, and they did not experience any further episodes of hypomagnesemia) at the fifth-month follow-up.
				2	Female	70	No DM	FBS (mg/dL): 90; Potassium (mEq/L): 4.2; Calcium (mg/dL): 8.8; Albumin (gm/dL): 4.3; Creatinine (mg/dL): 0.7	FBS (mg/dL): 83; Potassium (mEq/L): 4.6; Calcium (mg/dL): 8.9; Albumin (gm/dL): 3.7; Creatinine (mg/dL): 0.63	24-h urine output (mg): NA; FEMg: NA	Platinum-based chemotherapy and/or GI events	Patient was given oral magnesium gluconate 500 mg three tablets two times a day (total of 162 mg of elemental magnesium per day) and magnesium sulfate 2 gram IV three times per week, amiloride 10 mg twice a day	Dapagliflozin 10 mg/d	Serum magnesium levels were normal within three weeks and intravenous supplementation was stopped at 10 months of follow-up.
				3	Male	50	No DM	FBS (mg/dL): 99; Potassium (mEq/L): 3.3; Calcium (mg/dL): 9.6; Albumin (gm/dL): 4.1; Creatinine (mg/dL): 0.73	FBS (mg/dL): 104; Potassium (mEq/L): 4.1; Calcium (mg/dL): 8.9; Albumin (gm/dL): 4.1; Creatinine (mg/dL): 0.70	24-h urine output (mg): NA; FEMg: NA	Platinum-based chemotherapy and/or GI events	Patient was started on oral magnesium oxide 400 mg two tablets two times a day (total of 964 mg of elemental magnesium per day) and intermittent magnesium sulfate 2-4 gram IV, amiloride 10 mg two times a day.	Dapagliflozin 10 mg/d	Serum magnesium levels were normal and patient remained off intravenous magnesium supplementation along with at least two months of follow-up.
				4	Female	50	No DM	FBS (mg/dL): 80; Potassium (mEq/L): 4.1; Calcium (mg/dL): 9.2; Albumin (gm/dL): 3.9; Creatinine (mg/dL): 0.85	FBS (mg/dL): N.A.; Potassium (mEq/L): 4.0; Calcium (mg/dL): 9.7; Albumin (gm/dL): 4.0; Creatinine (mg/dL): 0.84	24-h urine output (mg): NA; FEMg: NA	Platinum-based chemotherapy and/or GI events	Patient was started on oral magnesium (magnesium 84 mg extended-release tablets two times a day), eplerenone 25 mg daily, magnesium sulfate 2 gram IV three times a week.	Dapagliflozin 10 mg/d	Improvement in serum magnesium levels with decreased requirement of IV MgSO_4_ requirement over one month of follow-up.
Shah et al. 2022 [11]	USA	Case report	2	1	Female	78	Type 2	HbA1c (%): 9.5; PTH (pg/mL): 40; 25-hydroxy vitamin D (ng/mL): 38; Calcium (mg/dL): 9.2; Albumin (mg/dL): 4.0; Creatinine (mg/dL): 0.80	HbA1c (%): 8.5; PTH (pg/mL): 63; 25-hydroxy vitamin D (ng/mL): 31; Calcium (mg/dL): 9.4; Albumin (mg/dL): 4.2; Creatinine (mg/dL): 0.98	24-hour urine Mg2+ (mg): Pre: 56; Post: 128; FEMg: Pre: 4.62%; Post: 8.37%	Chronic use of polyethylene glycol? for constipation	Patient was given oral magnesium, intravenous MgSO4 4 to 6 grams weekly.	Empagliflozin 10 mg/day	Taking empagliflozin 10 mg daily significantly improved her magnesium levels and allowed her to completely stop receiving magnesium sulfate (MgSO_4_) through IV and significantly reduce her daily oral magnesium glycinate supplements from 900 mg to 400 mg, she hasn't had any further magnesium deficiency episodes.
				2	Male	73	Type 2	HbA1c (%): 7.7; PTH (pg/mL): 37; 25-hydroxy vitamin D (ng/mL): 43; Calcium (mg/dL): 9.4; Albumin (mg/dL): 3.9; Creatinine (mg/dL): 1.2	HbA1c (%): 7.6; PTH (pg/mL): N.A.; 25-hydroxy vitamin D (ng/mL): N.A.; Calcium (mg/dL): 9.5; Albumin (mg/dL): N.A.; Creatinine (mg/dL): 1.2	24-hour urine Mg2+ (mg): Pre: 80; Post: 88; FEMg: Pre: 5.05%; Post: 4.27%	Not specified	Maximum tolerated oral magnesium, multiple ED visits requiring intravenous MgSO_4_	Empagliflozin 12.5 mg/day	After about a month of treatment, her magnesium levels returned back to normal, and her symptoms improved. This improvement lasted, and she was eventually able to lower the dose of her magnesium supplements.
Saha et al. 2022 [12]	USA	Case report	1	1	Female	58	Type 2	Serum Mg of 1.2 mg/dL, fractional excretion of Mg 8.9%, HbA1c: 7.9%	N.A.	N.A.	Excessive renal losses	Oral magnesium supplementation with intermittent requirement of intravenous infusions	Empagliflozin 10 mg daily.	Patient’s magnesium levels returned to normal within four weeks of treatment, along with an improvement in her symptoms. This positive change continued, allowing for a reduction in her daily magnesium supplement dosage.
Ray et al. 2020 [13]	USA	Case report	3	1	Male	60	Type 2	Magnesium (mmol/L): 1.4 ± 0.3; Sodium (mmol/L): 139 ± 3; Potassium (mmol/L): 4 ± 0.3; Calcium (mg/dL): 9 ± 0.6; Albumin (mg/dL): 4.1 ± 0.1; Creatinine (mg/dL): 1.1 ± 0.1	Magnesium (mmol/L): 1.9 ± 0.1; Sodium (mmol/L): 140 ± 1; Potassium (mmol/L): 4.4 ± 0.2; Calcium (mg/dL): 9.2 ± 0.3; Albumin (mg/dL): 4.1 ± 0.2; Creatinine (mg/dL): 1.0 ± 0.10	Creatinine 24 h: Pre: 1175; Post: 1220 ± 32; Magnesium 24 h: Pre: 325 ± 248; Post: 361 ± 21; Glucose 24 he: Pre: 0.07 ± 0.01; Post: 39	17q12 deletion	Maximum tolerated oral magnesium plus loperamide for loose stools; intravenous magnesium (5 g/d, split into AM and PM infusions); amiloride (10 mg 3×/d)	Canagliflozin (300 mg/d)	Within eight weeks, his mean serum magnesium level demonstrated statistically significant improvement.
				2	Female	60	Type 2	Magnesium (mmol/L): 0.9 ± 0.1; Sodium (mmol/L): 140 ± 2; Potassium (mmol/L): 4.3 ± 0.2; Calcium (mg/dL): 10.7 ± 0.5; Albumin (mg/dL): 3.8 ± 0.1; Creatinine (mg/dL): 0.88 ± 0.04	Magnesium (mmol/L): 1.2 ± 0.2; Sodium (mmol/L): 139 ± 1; Potassium (mmol/L): 4.7 ± 0.4; Calcium (mg/dL): 10.5 ± 0.2; Albumin (mg/dL): 3.8 ± 0.1; Creatinine (mg/dL): 1.03 ± 0.11	Creatinine 24 h: Pre: 1350 ± 99; Post: 1391 ± 126; Magnesium 24 h: 4.7 v 0.4. Pre: 127 ± 30; Post: 184 ± 18	Unknown	Maximum tolerated oral magnesium; Intravenous infusions previously discontinued due to transient efficacy; did not tolerate amiloride due to hyperkalemia	Empagliflozin (10 mg/d)	After taking empagliflozin for about five weeks, the patient's magnesium levels rose and were noticeable. They also needed less insulin each day, and the swelling in their lower legs improved.
				3	Female	20	Type 2	Magnesium (mmol/L): 1.2 ± 0.2; Sodium (mmol/L): 139 ± 2; Potassium (mmol/L): 4.1 ± 0.2; Calcium (mg/dL): 9.6 ± 0.3; Albumin (mg/dL): 3.8 ± 0.3; Creatinine (mg/dL): 83 ± 0.10	Magnesium (mmol/L): 1.5 ± 0.1; Sodium (mmol/L): 141 ± 2; Potassium (mmol/L): 4.6 ± 0.4; Calcium (mg/dL): 10.0; Albumin (mg/dL): 3.8; Creatinine (mg/dL): 1.15 ± 0.03	Creatinine 24 h: Pre: 1234 ± 212; Post: 1348 ± 80; Magnesium 24 h: Pre: 220 ± 132; Post: 138 ± 17; Glucose 24 h: Pre: 0.07 ± 0.01; Post: 50 ± 5	HNF1B deletion	Oral magnesium was given; intravenous magnesium was stopped due to recurrent deep vein thrombosis and superior vena cava syndrome	Dapagliflozin (10 mg/d)	Initiation of dapagliflozin therapy was associated with increased serum magnesium levels, reduced FEMg and improvement in muscle cramping and headaches.
Song et al. 2020 [14]	USA	Retrospective observational	50	N.A.	M: 66% F: 34%	57.03	Type 2	Magnesium: 1.6	Magnesium: 1.73	Treated UTI n (%): 7 (14)	Calcineurin inhibitors	N.A.	Empagliflozin (86%) Canagliflozin (12%) Dapagliflozin (2%)	Following a six-month observation period, patients taking empagliflozin showed a statistically significant average weight loss of 2.95 kilograms and an average improvement in low magnesium levels of 0.13.
Toto et al. 2019 [15]	Multi-centric	Post hoc analysis of 10 RCTs	4398	N.A.	M: 55.6% F: 44.4%	59.7	Type 2	In overall type 2 diabetes population: Serum magnesium levels: Adjusted change at week 24 from baseline Mean (95% CI): Placebo-0.00 (−0.01, 0.00), Dapagliflozin 10 mg-0.05 (0.05,0.06), Difference vs placebo (95% CI): 0.06 (0.05, 0.06), Hypomagnesemia: Serum magnesium-<0.74mmol/L, Adjusted change at week 24 from baseline Mean (95% CI): Placebo-0.05 (0.04, 0.06) Dapagliflozin 10 mg-0.13 (0.12,0.13), Difference vs placebo (95% CI): 0.08 (0.07, 0.09). Normal/hypermagnesemia: Serum magnesium-≥0.74 mmol/L, Adjusted change at week 24 from baseline Mean (95% CI): Placebo- -0.01 (-0.02, -0.01) Dapagliflozin 10 mg-0.04 (0.04, 0.04), Difference vs placebo (95% CI): 0.05 (0.05, 0.06)	N.A.	N.A.	Not specified	Magnesium supplementation	Dapagliflozin	Treatment with dapagliflozin 10 mg resulted in the correction of Mg concentrations in patients with T2D and hypomagnesemia.
Tang et al. 2016 [16]	Multi-centric	Meta-analysis	15,309	N.A.	Both male and female	58	Type 2	Canagliflozin 100 mg increased magnesium by 0.06 mmol/L, 300 mg by 0.09 mmol/L. Dapagliflozin 10 mg increased by 0.1 mmol/L. Empagliflozin 10 mg by 0.04 mmol/L, 25 mg by 0.07 mmol/L. Ipragliflozin 50 mg by 0.05 mmol/L	N.A.	N.A.	N.A.	N.A.	Canagliflozin 100 mg/300 mg Dapagliflozin 10 mg Empagliflozin 10 mg/25 mg Ipragliflozin 50 mg	SGLT2 inhibitors marginally increased serum magnesium levels in type 2 diabetes patients.

Overall, the evidence synthesized from these diverse study designs suggests that SGLT2 inhibitors may have a beneficial effect on serum magnesium levels in patients with T2DM and potentially in other conditions associated with hypomagnesemia. However, further research is needed to elucidate the underlying mechanisms and explore the clinical significance of these findings.

Discussion

The meta-analysis conducted by Tang et al. aimed to evaluate the impact of sodium-glucose co-transporter 2 inhibitors (SGLT2i) on serum electrolyte levels in patients with type 2 diabetes mellitus (T2DM). The researchers performed a comprehensive search across multiple databases to identify eligible parallel-design randomized controlled trials (RCTs) with a duration of at least 24 weeks, comparing SGLT2i with placebo in adults with T2DM. The primary outcome measure was the mean change from baseline in serum magnesium levels, while secondary outcomes included changes in serum sodium, phosphate, potassium, and calcium concentrations. The meta-analysis included 18 eligible RCTs, with a total of 15,309 participants, evaluating four different SGLT2i: canagliflozin, dapagliflozin, empagliflozin, and ipragliflozin. This analysis demonstrated that SGLT2 inhibitor administration in patients without chronic kidney disease resulted in a statistically significant elevation of serum magnesium levels compared to a placebo control group. Notably, canagliflozin demonstrated a dose-dependent linear association with increased magnesium levels. Furthermore, dapagliflozin was found to significantly increase serum phosphate levels, while serum sodium levels varied depending on the specific SGLT2i used, and there were no significant changes observed in potassium and calcium levels. The authors concluded that SGLT2i marginally increased serum magnesium levels in T2DM patients, highlighting the need for further studies to assess the clinical significance of this observation [16].

In 2020, Ray et al. reported a case series involving three patients, shedding light on the potential impact of SGLT2 inhibitors on magnesium levels. The findings suggested that these inhibitors could play a role in improving magnesium levels, particularly in individuals with urinary magnesium wasting. All three patients exhibited a statistically significant rise in serum magnesium levels within an eight-week timeframe. Notably, more frequent monitoring intervals could have potentially expedited the attainment of statistical significance. SGLT2 inhibitor therapy was associated with a mean increase of 0.36 ± 0.12 mg/dL in serum magnesium concentrations across the three patients during periods excluding routine intravenous supplementation. This case series provided valuable insights into the potential benefits of SGLT2 inhibitors in managing hypomagnesemia [13].

In 2022, Saha et al*.* reported a case where SGLT2 inhibitor therapy led to a rapid improvement in serum magnesium levels, reaching 2.2 mg/dL within four weeks. The sustained improvement on subsequent evaluations, coupled with reduced symptoms and a decreased oral magnesium supplement dose, suggested a positive therapeutic impact [12].

The fractional excretion of magnesium (FEMg) assesses renal magnesium handling and helps differentiate causes of hypermagnesemia and hypomagnesemia. A low FEMg suggests efficient renal reabsorption, while a high FEMg indicates impaired reabsorption or increased excretion. It is calculated using the formula:

FEMg (%) = (Urine Magnesium×Plasma Creatinine Plasma Magnesium×Urine Creatinine) × 100FEMg (%) =(Plasma Magnesium×Urine Creatinine Urine Magnesium×Plasma Creatinine​) × 100.

In a 2022 case report by Shah, two cases were presented that challenged the conventional understanding of SGLT2 inhibitors in hypomagnesemia. Both individuals presented with refractory hypomagnesemia and were reliant on high-dose maintenance magnesium replacement therapy. A noteworthy observation was that despite the absence of a significant improvement in urinary magnesium wasting, as indicated by fractional excretion of magnesium (FEMg) before and after treatment, both patients experienced a correction of their hypomagnesemia. This finding challenged the prevailing notion that the therapeutic efficacy of SGLT2 inhibitors in hypomagnesemia primarily stems from their impact on urinary magnesium wasting. Additionally, the study highlighted low 24-hour urinary magnesium levels, measuring 56 mg in case 1 and 80 mg in case 2. These levels were deemed insufficient to attribute the severity of refractory hypomagnesemia solely to a kidney-related etiology. Despite the low urinary magnesium levels, FEMg values were slightly above the expected range for nonrenal causes of hypomagnesemia, recording 4.62% for case 1 and 5.05% for case 2. The report suggested that the severe and refractory hypomagnesemia in these cases could be influenced by both renal and nonrenal factors, emphasizing the intricate nature of hypomagnesemia etiology [11].

A separate report by Shah (2023) underscored the potential benefits of SGLT2 inhibitors in managing severe hypomagnesemia, even in non-diabetic patients. Leveraging data from randomized controlled trials and existing case reports, the study identified an elevation of serum magnesium levels as a class effect of SGLT2 inhibitors in individuals with type 2 diabetes. Notably, the study discussed cases where SGLT2 inhibitors demonstrated significant benefits in correcting refractory hypomagnesemia, even in patients without overt urinary magnesium wasting. The etiology of hypomagnesemia varied among the cases, including urinary magnesium wasting due to calcineurin inhibitors in one case and potential platinum-based chemotherapy-related causes in three other cases. The report explored potential mechanisms of SGLT2 inhibitors on magnesium balance, considering factors like increased glucagon and arginine vasopressin secretion. While the exact mechanisms remain an area for further investigation, the study suggested that SGLT2 inhibitors could have extraglycemic benefits, including improved magnesium balance, and supported the need for expanded research to better understand their effects in a larger patient population [10].

Toto et al*.* conducted a post-hoc analysis involving 10 clinical trials with over 4000 participants, providing a comprehensive evaluation of the impact of dapagliflozin 10 mg on serum magnesium concentrations in individuals with type 2 diabetes (T2D). The study reported a prevalence of hypomagnesemia in T2D patients (17.6%), consistent with previously documented ranges (14%-48%). Patients with baseline hypomagnesemia exhibited a higher incidence of cardiovascular issues, including heart disease, heart failure, hypertension, dyslipidemia, and peripheral arterial disease/peripheral vascular disease, aligning with existing literature. Dapagliflozin 10 mg demonstrated a significantly greater mean change in serum magnesium compared to placebo from baseline to week 24, particularly pronounced in patients with baseline hypomagnesemia. The treatment also led to a higher proportion of patients with hypomagnesemia at baseline achieving serum magnesium levels within the normal range after 24 weeks. Although hypermagnesemia rates were low, dapagliflozin 10 mg effectively reduced both systolic and diastolic blood pressure without significant differences between serum magnesium subgroups. This suggests that the blood pressure-lowering effects of dapagliflozin in T2D patients remain unaffected by hypomagnesemia [15].

In a 2020 retrospective single-center study by Song, the focus was on adult kidney transplant (KT) recipients meeting specific criteria for initiating SGLT-2 inhibitors. The eligible patient cohort included those with type II diabetes, an absence of acute kidney injury within the prior 30 days, no urinary tract infections in the preceding six months, and an estimated glomerular filtration rate (eGFR) ≥30 mL/min at initiation. The study evaluated outcomes, such as changes in weight, insulin dosage, HbA1C, magnesium concentration, and safety parameters, in a group of 50 patients meeting the inclusion criteria. Noteworthy findings included significant improvements in weight (−2.95 kg) and magnesium concentration (0.13) over a mean follow-up of 101 days. While overall insulin requirements decreased, the difference lacked statistical significance. The study reported no instances of diabetic ketoacidosis, amputations, or acute kidney injury episodes, but 14% of patients developed urinary tract infections. Nine patients discontinued therapy, primarily due to urinary tract infections. Despite indicating a non-significant short-term impact on diabetic management, the study suggested the safe utilization of SGLT-2 inhibitors in post-transplant diabetes management, potentially addressing metabolic complications and electrolyte abnormalities linked to prolonged immunosuppression [14].

SGLT2 inhibitors exert a multifaceted influence on magnesium reabsorption. Their primary mechanism involves reducing electrogenic sodium-glucose cotransport in the proximal tubule. This action elevates the intraluminal electrical potential, subsequently promoting magnesium reabsorption [17]. Additionally, SGLT2 inhibitor therapy induces extracellular fluid volume depletion, leading to the activation of the renin-angiotensin-aldosterone system (RAAS). RAAS activation is thought to contribute to tubular magnesium uptake, possibly via solvent drag [18].

Beyond these established mechanisms, several other potential contributors to the observed increase in magnesium reabsorption with SGLT2 inhibitors have been proposed. These include alterations in the expression of renal or gut magnesium transporters, potential hypertrophy or hyperplasia of tubular segments involved in magnesium transport, and changes in the production of signaling molecules like epidermal growth factor that may influence magnesium reabsorption [19].

The impact of SGLT2 inhibition on magnesium reabsorption may extend beyond its direct influence on sodium and glucose transport. While glucagon is known to promote tubular magnesium reabsorption, evidence suggests minimal changes in glucagon levels with SGLT2 inhibitor use [20]. Conversely, insulin may also stimulate tubular magnesium uptake. Improved glycemic control achieved with SGLT2 inhibitors could potentially lead to better insulin sensitivity and subsequently, increased tubular magnesium reabsorption. However, the observed improvements in magnesium handling are likely not solely attributable to altered insulin or glucagon signaling [21,22].

Reduced glomerular filtration of magnesium could be another contributing factor to decreased urinary magnesium loss. SGLT2 inhibitor-induced reductions in glomerular filtration rate, attributed to enhanced tubuloglomerular feedback, may reduce both afferent and efferent arteriolar tone, resulting in decreased glomerular filtration. However, the study faced challenges in assessing changes in magnesium filtration due to unstable baseline glomerular filtration rates [23].

This study raises an intriguing possibility: the therapeutic potential of SGLT2 inhibitors for managing hypomagnesemia in non-diabetic patients. The DAPA-HF trial, which evaluated dapagliflozin in a predominantly non-diabetic heart failure population, provides evidence for the safety of SGLT2 inhibitors in this group [24]. However, further well-designed studies are warranted to definitively clarify the impact of SGLT2 inhibitors on serum magnesium levels and urinary magnesium excretion in this specific patient population.

Limitations

The study has several notable limitations that must be acknowledged. Firstly, the inclusion of a limited number of studies, some with small effect sizes, encompasses diverse research methodologies such as case reports, retrospective observational studies, and post-hoc analyses of randomized controlled trials (RCTs). This methodological variety introduces challenges in terms of generalizability and statistical robustness of the findings. This analysis is further limited by the non-randomized allocation of interventions across the included studies. Additionally, the absence of a standardized protocol for diagnosing and treating hypomagnesemia with SGLT2 inhibitors introduces potential inconsistencies in patient management and outcome assessment. These factors heighten the risk of selection bias and hinder the establishment of a clear cause-and-effect relationship between SGLT2 inhibitor use and improvements in hypomagnesemia. Moreover, the inherent heterogeneity across treatment settings and the limited availability of data further impede a comprehensive and conclusive analysis of this topic. The diverse contexts in which the studies were conducted may limit the ability to draw overarching conclusions applicable to all patient populations and clinical scenarios.

To strengthen the reliability and applicability of the conclusions drawn from this systematic review, it is imperative to conduct further research that addresses these limitations. Larger-scale, well-designed randomized controlled trials with standardized protocols for intervention and assessment are crucial to providing more robust evidence on the impact of SGLT2 inhibitors on hypomagnesemia across various patient populations. Such rigorous studies would help mitigate potential biases and establish clearer causal links between the intervention and outcomes of interest.

## Conclusions

This systematic review highlights the potential effectiveness of SGLT2 inhibitors in addressing refractory hypomagnesemia, particularly in cases associated with urinary magnesium wasting. The findings from the included studies suggest that SGLT2 inhibitor therapy may lead to significant improvements in serum magnesium levels and correction of hypomagnesemia, even in patients with long-standing and treatment-resistant cases. Further exploration of the mechanisms underlying the magnesium-modulating effects of SGLT2 inhibitors may pave the way for novel therapeutic strategies and personalized approaches to maintaining magnesium homeostasis.

## Appendices

Search strategies

Web of Science

(Sodium-Glucose Transporter 2 Inhibitors OR SGLT-2 OR SGLT2 OR empagliflozin OR tofogliflozin OR canagliflozin OR dapagliflozin OR luseogliflozin OR sotagliflozin OR remogliflozin OR ipragliflozin OR ertugliflozin OR sergliflozin) AND (Hypomagnesemia OR magnesium OR magnesium supplementation).

PubMed

("Sodium-Glucose Transporter 2 Inhibitors"(Mesh) OR "SGLT2"(tw) OR "SGLT-2"(tw) OR "SGLT 2*"(tw) OR "tofogliflozin"(tw) OR "empagliflozin"(tw) OR "dapagliflozin"(tw) OR "canagliflozin"(tw) OR "sotagliflozin"(tw) OR "luseogliflozin"(tw) OR "ipragliflozin"(tw) OR "remogliflozin"(tw) OR "sergliflozin"(tw) OR "ertugliflozin"(tw)) AND (“Hypomagnesemia”(Mesh)” OR “Magnesium”(tw) OR “Magnesium supplementation”(tw)).

Scopus

(“Sodium-Glucose cotransporter 2 Inhibitors” OR SGLT2 OR SGLT-2” OR empagliflozin OR tofogliflozin OR canagliflozin OR ertugliflozin OR sotagliflozin OR dapagliflozin OR ipragliflozin OR luseogliflozin OR sergliflozin OR remogliflozin) AND (“Hypomagnesemia” OR “magnesium” OR “magnesium supplementation”).

Google Scholar

Sodium-glucose co-transporter 2 inhibitors + Hypomagnesemia

**Table 2:** Quality assessment of included case reports using CARE guidelines. CARE: CAse REport.

Reference	Keywords	Abstract	Introduction	Patient info	Clinical findings	Timeline	Diagnostic intervention	Therapeutic intervention	Follow-up and outcomes	Discussion	Patient perspective	Informed consent
Ray et al. 2020 [13]	No	Yes	Yes	Yes	Yes	Yes	Yes	Yes	Yes	Yes	No	Yes
Saha et al. 2022 [12]	Yes	Yes	Yes	Yes	Yes	Yes	Yes	Yes	Yes	Yes	No	Yes
Shah et al. 2022 [11]	Yes	Yes	Yes	Yes	Yes	Yes	Yes	Yes	Yes	Yes	No	Yes
Shah et al. 2023 [10]	Yes	Yes	Yes	Yes	Yes	Yes	Yes	Yes	Yes	Yes	No	Yes

**Table 3:** Quality assessment of included observational study using STROBE guidelines. STROBE: Strengthening the Reporting of Observational Studies in Epidemiology. Reference: [14].

	Item no	Recommendation	Y/N (page no)
Title and abstract	1	(a) Indicate the study’s design with a commonly used term in the title or the abstract	Y (1)
		(b) Provide in the abstract an informative and balanced summary of what was done and what was found	Y (1)
Introduction			
Background/rationale	2	Explain the scientific background and rationale for the investigation being reported	Y (1)
Objectives	3	State specific objectives, including any prespecified hypotheses	Y (1)
Methods			
Study design	4	Present key elements of study design early in the paper	Y (1)
Setting	5	Describe the setting, locations, and relevant dates, including periods of recruitment, exposure, follow-up, and data collection	Y (1)
Participants	6	(a) Cohort study—Give the eligibility criteria, and the sources and methods of selection of participants. Describe methods of follow-up Case-control study—Give the eligibility criteria, and the sources and methods of case ascertainment and control selection. Give the rationale for the choice of cases and controls Cross-sectional study—Give the eligibility criteria, and the sources and methods of selection of participants	N
		(b) Cohort study—For matched studies, give matching criteria and number of exposed and unexposed Case-control study—For matched studies, give matching criteria and the number of controls per case	N
Variables	7	Clearly define all outcomes, exposures, predictors, potential confounders, and effect modifiers. Give diagnostic criteria, if applicable	Y (1)
Data sources/measurement	8	For each variable of interest, give sources of data and details of methods of assessment (measurement). Describe comparability of assessment methods if there is more than one group	N
Bias	9	Describe any efforts to address potential sources of bias	N
Study size	10	Explain how the study size was arrived at	Y (2)
Quantitative variables	11	Explain how quantitative variables were handled in the analyses. If applicable, describe which groupings were chosen and why	Y (2)
Statistical methods	12	(a) Describe all statistical methods, including those used to control for confounding	Y (2)
		(b) Describe any methods used to examine subgroups and interactions	NA
		(c) Explain how missing data were addressed	NA
		(d) Cohort study—If applicable, explain how loss to follow-up was addressed Case-control study—If applicable, explain how matching of cases and controls was addressed Cross-sectional study—If applicable, describe analytical methods taking account of sampling strategy	NA
		(e) Describe any sensitivity analyses	NA
Results			
Participants	13	(a) Report numbers of individuals at each stage of study—e.g., numbers potentially eligible, examined for eligibility, confirmed eligible, included in the study, completing follow-up, and analysed	Y (2)
		(b) Give reasons for non-participation at each stage	N
		(c) Consider use of a flow diagram	N
Descriptive data	14	(a) Give characteristics of study participants (e.g., demographic, clinical, social) and information on exposures and potential confounders	Y (2)
		(b) Indicate number of participants with missing data for each variable of interest	N
		(c) Cohort study—Summarixe follow-up time (e.g., average and total amount)	NA
Outcome data	15	Cohort study—Report numbers of outcome events or summary measures over time	Y (2)
		Case-control study—Report numbers in each exposure category, or summary measures of exposure	NA
		Cross-sectional study—Report numbers of outcome events or summary measures	NA
Main results	16	(a) Give unadjusted estimates and, if applicable, confounder-adjusted estimates and their precision (e.g., 95% confidence interval). Make clear which confounders were adjusted for and why they were included	Y (2)
		(b) Report category boundaries when continuous variables were categorized	N
		(c) If relevant, consider translating estimates of relative risk into absolute risk for a meaningful time period	N
Other analyses	17	Report other analyses done—e.g., analyses of subgroups and interactions, and sensitivity analyses	N
Discussion			
Key results	18	Summarize key results with reference to study objectives	Y (2)
Limitations	19	Discuss limitations of the study, taking into account sources of potential bias or imprecision. Discuss both direction and magnitude of any potential bias	Y (2)
Interpretation	20	Give a cautious overall interpretation of results considering objectives, limitations, multiplicity of analyses, results from similar studies, and other relevant evidence	Y (2)
Generalizability	21	Discuss the generalizability (external validity) of the study results	N
Other information			
Funding	22	Give the source of funding and the role of the funders for the present study and, if applicable, for the original study on which the present article is based	N
